# Mammographic density in relation to breast cancer recurrence and survival in women receiving neoadjuvant chemotherapy

**DOI:** 10.3389/fonc.2023.1177310

**Published:** 2023-06-14

**Authors:** Anna Zdanowski, Hanna Sartor, Maria Feldt, Ida Skarping

**Affiliations:** ^1^ The Faculty of Medicine, Lund University, Lund, Sweden; ^2^ Department of Translational Medicine, Diagnostic Radiology, Skåne University Hospital, Lund University, Lund/Malmö, Sweden; ^3^ Division of Oncology, Department of Clinical Sciences, Lund University, Lund, Sweden; ^4^ Department of Oncology, Skåne University Hospital, Lund, Sweden; ^5^ Department of Clinical Physiology and Nuclear Medicine, Skåne University Hospital, Lund, Sweden

**Keywords:** survival analysis (MeSH NLM), breast cancer neoadjuvant therapy, mammographic density (MD), breast density, imaging

## Abstract

**Objective:**

The association between mammographic density (MD) and breast cancer (BC) recurrence and survival remains unclear. Patients receiving neoadjuvant chemotherapy (NACT) are in a vulnerable situation with the tumor within the breast during treatment. This study evaluated the association between MD and recurrence/survival in BC patients treated with NACT.

**Methods:**

Patients with BC treated with NACT in Sweden (2005–2016) were retrospectively included (N=302). Associations between MD (Breast Imaging-Reporting and Data System (BI-RADS) 5^th^ Edition) and recurrence-free/BC-specific survival at follow-up (Q1 2022) were addressed. Hazard ratios (HRs) for recurrence/BC-specific survival (BI-RADS a/b/c vs. d) were estimated using Cox regression analysis and adjusted for age, estrogen receptor status, human epidermal growth factor receptor 2 status, axillary lymph node status, tumor size, and complete pathological response.

**Results:**

A total of 86 recurrences and 64 deaths were recorded. The adjusted models showed that patients with BI-RADS d vs. BI-RADS a/b/c had an increased risk of recurrence (HR 1.96 (95% confidence interval (CI) 0.98–3.92)) and an increased risk of BC-specific death (HR 2.94 (95% CI 1.43–6.06)).

**Conclusion:**

These findings raise questions regarding personalized follow-up for BC patients with extremely dense breasts (BI-RADS d) pre-NACT. More extensive studies are required to confirm our findings.

## 1 Introduction

Breast cancer (BC) is the most common malignancy among women (1). Recurrence and survival rates vary among BC subtypes, with triple-negative BC [negative estrogen receptor (ER), progesterone receptor (PR), and Human Epidermal Growth Factor Receptor 2 (HER2)] as the most aggressive subtype (2). Metastatic disease is considered to be incurable, with a 10-year survival rate of only 13% (3).

Mammographic density (MD) is determined based on the amount of radiopaque breast parenchyma in the mammogram and describes the relationship between the white dense breast tissue and dark transparent fatty tissue (4). MD is established as one of the strongest risk factors for BC after BC gene mutations and age (5). In a meta-analysis by McCormack et al. (6), based on 42 studies, it was concluded that women with high MD (>75% dense tissue) had a 4- to 6-fold increased risk of BC compared to women with low MD (<5% dense tissue). Moreover, the sensitivity of mammographic BC screening is negatively affected by MD, particularly for women with extremely dense breasts (7).

MD varies throughout life but declines with age, with the biggest drop occurring during the shift to menopause (8). Low MD is associated with older age, higher body mass index (BMI), multiple pregnancies, and parity, whereas high MD is associated with younger age, lower BMI, nulliparity, older age at first birth, and use of hormone replacement therapy (9–11). It is unknown whether high MD leads to more aggressive BC forms (12), as it is unrelated to BRCA mutations (13), tumor hormone receptor status, or BC phenotypes (5).

Most women with early BC are eligible for primary surgery. Systemic chemotherapy is often administered in relation to surgery, either neoadjuvant or adjuvantly (14), with both methods having comparable survival and recurrence rates (15). Neoadjuvant chemotherapy (NACT) is the standard treatment for patients with HER2 positive BC, triple-negative BC, and patients presenting with an inoperable primary tumor (16); this has enabled conversion from mastectomy to less extensive partial mastectomy (17). Additionally, NACT allows for monitoring of the tumor treatment response *in vivo* (18), allowing for the assessment of potential accomplishment of complete pathological response (pCR), which helps guide the adjuvant therapy choice. However, further predictive markers are needed to avoid under- and over-treatment.

Studies, among one by our research group (19), have suggested that extremely dense breasts (Breast Imaging-Reporting and Data System (BI-RADS) d) pre-NACT (baseline) decreases the chances of achieving pCR post-NACT, suggesting that MD could be used as a predictive marker of pCR (20, 21). However, a recent study by Di Cosimo et al. showed that patients with mammographically dense breasts were more likely to attain pCR (BIRADS a vs. b/c/d) (22), whereas Cullinane et al. found no association between MD and pCR (23). Similar to our observations, some also reported that MD decreases after NACT, most likely due to structural changes and temporary menopause caused by treatment (24, 25).

There is a lack of consensus regarding the association between MD and BC recurrence or survival (26). Eriksson et al. (27) found that MD was linked to local and locoregional recurrence but not with distant recurrence or survival. Concurrently, Heindl et al. (28) carried out a more extensive cohort study (N=2,525), showing no association between MD and disease-free or overall survival. The association between baseline MD and BC events following NACT has not been extensively studied (20, 29, 30). Women with BC who receive NACT are in a vulnerable situation, with tumors in the breast during treatment, often with more advanced BC, and thus have a worse prognosis. Therefore, there is an urgent need for more predictive markers, including imaging biomarkers for NACT-treated patients, to improve and individualize treatment.

This study aimed to evaluate the association between MD at baseline (assessed using the BI-RADS) and BC recurrence and BC-specific survival in a retrospective cohort of patients treated with NACT, concomitant to generate more information about treatment response and prognosis.

## 2 Methods

**Figure 1:**
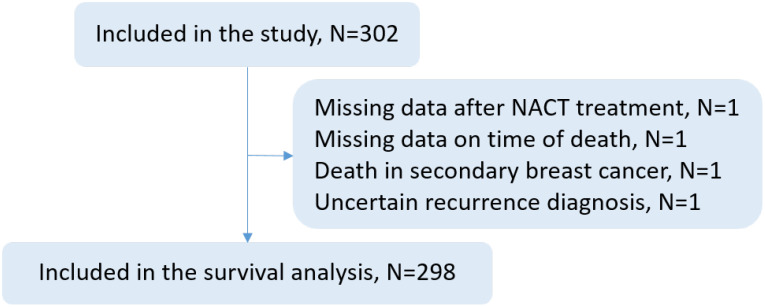
Flowchart describing the number (N) of patients included in the study: 302 patients who received neoadjuvant chemotherapy (NACT) for breast cancer between 2005 and 2016 at Skane University Hospital, Sweden, were included in the study, and 298 patients were included in the survival analyses. Four patients were excluded from survival analyses due to lack of data, unclear circumstances surrounding recurrence or death, or death in secondary breast cancer.

Female BC patients treated with NACT in Sweden from 2005-2016 [NACT treatment according to the national guidelines (16)], were retrospec included (N=302). Cohort baseline data have been previously described (16, 19) and are briefly recapitulated here. Exclusion criteria included male patients (N=2), bilateral BC at diagnosis (N=8), not undergoing planned surgery (N=23), misclassified NACT (N=42), lacking mammograms at diagnosis (N=34), and unwillingness to participate (N=8) [Figure 1 in (19)].

One radiologist blindly assessed the pre-NACT digital mammograms (all views, both breasts) and assigned each patient to one of four categories according to BI-RADS 5^th^ Edition for MD. The categories ranging from a to d, were a: breasts are “almost entirely fatty,” b: breasts have “scattered areas of fibroglandular density,” c: breasts are “heterogeneously dense,” and d: breasts are “extremely dense” (4).

Data on patient and tumor characteristics, NACT treatment, and surgery were collected from the clinical records in 2016. If the menopausal status was unknown, patients aged 55 years or older were assumed to be postmenopausal. Tumor size were measured before NACT in mammograms or ultrasound images; when both methods were conclusive, an average measure was used. The immunohistochemical profile based on core needle biopsies at the time of diagnosis and the criteria for positive immunohistochemistry were as follows: >10% for ER, >20% for Ki67, and 3+ for HER2 with immunochemistry and/or amplification with fluorescence and *in situ* hybridization. For pCR to be reached, no remaining invasive cancer is found in the resected breast or any sampled regional lymph nodes after completion of NACT (31). Upon final breast surgery, all patients had tumor-free margins according to clinical pathology reports.

In Q1 2022, the medical charts were reviewed again for subsequent BC events after surgery, such as recurrence (local and distant) and BC-specific survival. Furthermore, postsurgical characteristics of radiation, endocrine, adjuvant chemotherapy, and adjuvant HER2-targeted treatment were collected. Only verified recurrences and/or deaths due to primary BC were considered as events. A new BC was not considered a recurrence, and deaths due to new BC were not reason for censoring. Patients who moved out of the regional council area and thus had their medical charts obscured were censored.

Four patients were excluded from the survival analyses due to missing data after NACT treatment, uncertain recurrence/death status, or death in secondary BC (**Figure 1**). In the multivariate-adjusted Cox regression models, an additional 19 patients were excluded from the survival analyses due to missing values.

### 2.1 Rational for BI-RADS grouping strategy

Recognizing the distinct entity of BI-RADS d, in 2022, Bodewes et al. published a systematic review and meta-analysis dedicated to the association between MD BI-RADS d and BC risk, finding an approximately two-fold increased risk of BC for women with BI-RADS d compared to the general population (32). Recently, the European Society of Breast Imaging (EUSOBI) published separate screening recommendations for women with extremely dense breasts (BI-RADS d) due to a high level of evidence in this specific subgroup of women (33, 34), indicating the need to acknowledge the differences between BI-RADS c and d. From a biological perspective, we purposely chose to analyze the patients with the highest MD separately (BI-RADS d) and hypothesized that these women might harbor pro-carcinogenic breast tissue, which is more prone to both BC initiation and progression/recurrence. In addition, Rojas et al. (20) performed the same grouping in their study of MD and its association with disease free survival, thus facilitating the comparison of study results. Moreover, the inter-radiologist agreement is most significant for radiologists assessing the least dense (BI-RADS a) and most dense (BI-RADS d) categories of MD (35, 36), thus supporting the high validity of the MD scoring for these groups. Furthermore, to explore potential differences between the two high MD categories, BI-RADS c and d, as a supplementary analysis, these categories were analyzed separately with the two low MD categories, BI-RADS a and b, as reference (merged due to a few events in the BI-RADS a group).

### 2.2 Ethical approval

All procedures performed in this study involving human participants were in accordance with the ethical standards of the institutional and/or national research committee and with the 1964 Helsinki Declaration and its later amendments or with comparable ethical standards. The study was approved by the Regional Ethics Committee of Lund, Sweden (Reference No.: 2014/13 and 2016/521).

### 2.3 Statistical analyses

Baseline patient and treatment characteristics, as well as subsequent events, are presented in descriptive tables according to pre-NACT MD assessed using BI-RADS. Variables were summarized as either counts and percentages for categorical variables or as medians and interquartile ranges (IQRs) for continuous variables. The chi-square test and Kruskal–Wallis test, respectively, were used to calculate *P* values.

Survival analyses using Kaplan–Meier (KM) curves and Cox regression models were performed to address the association between MD at diagnosis and BC recurrence and BC-specific death. Analyses were performed using both BI-RADS a/b/c and BI-RADS a/b as references. Time variables were computed, depicting the time from primary BC diagnosis to either a BC event (first recurrence or BC-specific death) or the end of follow-up. KM curves with log-rank test results were generated to visualize recurrence-free survival and BC-specific survival among different BI-RADS groups. Initially, KM curves for the entire cohort were made and then split according to pCR. Cox regression analysis was used to calculate hazard ratios (HRs) with 95% confidence intervals (CIs). Crude and multivariate-adjusted models were constructed, and adjustments were made according to potential confounders that may influence prognosis, treatment response, and MD, such as age at diagnosis (years), tumor size (mm, mammography), pCR status (yes/no), ER (positive/negative), HER2 (positive/negative), and axillary lymph node status (N0/N+). *P* values should be interpreted as evidence against the null hypothesis of no association without reference to a threshold for significance (37, 38). All analyses were performed using IBM SPSS Statistics for Windows version 27 (IBM Corp., Armonk, N.Y., USA).

## 3 Results

### 3.1 Descriptive results

#### 3.1.1 Patient, tumor, and treatment characteristics

All 302 patients were included in the descriptive statistics analysis. The pre-NACT (baseline) data are presented in **Table 1**. Patients categorized as BI-RADS d were younger at diagnosis, had a lower BMI, were more often premenopausal, and their tumors were more frequently ER/PR-positive. Furthermore, there were no differences in tumor size, nodal status, proliferation (Ki67), or HER2 status. Administered NACT treatment was similar for patients between the MD groups. Most patients did not achieve pCR (N=232), with no significant difference between the BI-RADS groups. The adjuvant treatment is outlined in **Supplementary Material 1**.

**Table 1:** Patient and tumor characteristics at diagnosis, according to mammographic density, assessed with Breast Imaging-Reporting and Data System (BI-RADS).

		BI-RADS a	BI-RADS b	BI-RADS c	BI-RADS d	*P* value
Number of patients		16	120	140	26	
Age	Median (IQR)	59 (54–68)	59 (50–66)	49 (41–60)	44 (37–54)	<0.001*
BMI	Median (IQR)	30 (27–35)	27 (24–30)	24 (22–27)	23 (21–26)	<0.001*
Menopausal status	Premenopausal	6 (37.5)	33 (27.5)	81 (57.9)	17 (65.4)	<0.001**
	Postmenopausal	10 (62.5)	87 (72.5)	59 (42.1)	9 (34.6)	
Neoadjuvant chemotherapy	FEC/EC + taxane	15 (93.8)	98 (81.7)	110 (78.6)	20 (76.9)	0.812**
	Taxanes	1 (6.3)	16 (13.3)	25 (17.9)	5 (19.2)	
	FEC/EC		3 (2.5)	4 (2.9)	1 (3.8)	
	Other		3 (2.5)	1 (0.7)		
Neoadjuvant HER2 therapy	Yes	4 (25.0)	44 (36.7)	38 (27.1)	9 (34.6)	0.369**
	No	12 (75.0)	76 (63.3)	102 (72.9)	17 (65.4)	
Surgery method	Mastectomy	12 (75.0)	92 (76.7)	115 (82.1)	24 (92.3)	0.744**
	Sector	4 (25.0)	25 (20.8)	22 (15.7)	2 (7.7)	
	Mastectomy + sector		2 (1.7)	2 (1.4)		
	Missing		1 (0.8)	1 (0.7)		
pCR (surgical specimen post-NACT)	Yes	5 (31.3)	35 (29.2)	27 (19.3)	3 (11.5)	0.104**
	No	11 (68.8)	85 (70.8)	113 (80.7)	23 (88.5)	
Core needle biopsy: estrogen receptor status	Positive	5 (31.3)	69 (57.5)	89 (63.6)	20 (76.9)	0.018**
	Negative	11 (68.8)	47 (39.2)	46 (32.9)	6 (23.1)	
	Missing		4 (3.3)	5 (3.6)		
Core needle biopsy: progesterone receptor status	Positive	4 (25.0)	44 (36.7)	77 (55.0)	15 (57.7)	0.004**
	Negative	12 (75.0)	72 (60.0)	58 (41.4)	11 (42.3)	
	Missing		4 (3.3)	5 (3.6)		
Core needle biopsy: HER2 status	Positive	3 (18.8)	45 (37.5)	38 (27.1)	9 (34.6)	0.273**
	Negative	11 (68.8)	68 (56.7)	95 (67.9)	17 (65.4)	
	Missing	2 (12.5)	7 (5.8)	7 (5.0)		
Core needle biopsy: Ki67	>20% (high)	12 (75.0)	89 (74.2)	89 (63.6)	15 (57.7)	0.159**
	<=20% (low)	2 (12.5)	9 (7.5)	25 (17.9)	3 (11.5)	
	Missing	2 (12.5)	22 (18.3)	26 (18.6)	8 (30.8)	
Axillary node status (assessed with FNA or SLNB)	N0	5 (31.3)	30 (25.0)	37 (26.4)	9 (34.6)	0.689**
	N+	10 (62.5)	88 (73.3)	102 (72.9)	16 (61.5)	
	Missing	1 (6.3)	2 (1.7)	1 (0.7)	1 (3.8)	
Tumor size at diagnosis (mm)	Median (IQR)	34 (23–40)	30 (21–40)	35 (25–50)	30 (20–40)	0.122*

*Kruskal Wallis tes.t

**Chi-square test.

EC, Epirubicin and Cyclophosphamide; FEC, Fluorouracil, Epirubicin and Cyclophosphamide; FNA, fine-needle aspiration; HER2, human epidermal growth factor receptor 2; IQR, interquartile range; mast, mastectomy; mm, millimeter; pCR, complete pathological response; SLNB, sentinel lymph node biopsy.

#### 3.1.2 Recurrence and breast cancer-specific survival

A total of 86 and 64 events of BC recurrence and BC-specific death, respectively, were observed in the cohort (**Table 2**). The highest recurrence rate (42.3%, N=11) and the shortest time to recurrence [median of 1.9 years (IQR 1.3–4.1)] were seen in patients with extremely dense breasts (BI-RADS d). This group had the highest rate of BC-specific deaths, 38.5% (N=10), and the shortest time of survival [3.4 years (IQR 2.4–6.9)] vs. patients with lower MD.

**Table 2:** Breast cancer events after completed neoadjuvant chemotherapy according to mammographic density assessed with Breast Imaging-Reporting and Data System (BI-RADS) at diagnosis.

		BI-RADS a	BI-RADS b	BI-RADS c	BI-RADS d	*P* value
Number of patients		16	120	140	26	
Status	Alive	13 (81.3)	87 (72.5)	114 (81.4)	14 (53.8)	0.043*
	Primary breast cancer death	2 (12.5)	27 (22.5)	25 (17.9)	10 (38.5)	
	Death, other	1 (6.3)	5 (4.2)	1 (0.7)	2 (7.7)	
	Missing		1 (0.8)			
New breast cancer	Yes	2 (12.5)	2 (1.7)	4 (2.9)		0.067*
	No	14 (87.5)	117 (97.5)	136 (97.1)	26 (100)	
	Missing		1 (0.8)			
Recurrence	Yes	5 (31.3)	33 (27.5)	37 (26.4)	11 (42.3)	0.425*
	No	11 (68.8)	85 (70.8)	103 (73.6)	15 (57.7)	
	Missing		2 (1.7)			
Age at recurrence	Median (IQR)	61 (61–74)	63 (51–71)	52 (43–63)	49 (42–60)	0.017**
Time to recurrence (years)	Median (IQR)	5.11 (2.38–8.40)	2.34 (1.60–5.09)	2.80 (1.53–5.67)	1.90 (1.30–4.08)	0.313**
Recurrence location	Local			3 (2.1)		0.212*
	Locoregional	1 (6.3)	2 (1.7)		1 (3.8)	
	Distant	4 (25.0)	31 (25.8)	34 (24.3)	10 (38.5)	
	No	11 (68.8)	85 (70.8)	103 (73.6)	15 (57.7)	
	Missing		2 (1.7)			
Age at breast cancer death	Median (IQR)	63^***^	64 (53–72)	53 (44–69)	51 (42–61)	0.216**
Time to breast cancer death (years)	Median (IQR)	4.76^c^	3.89 (2.10–5.29)	4.21 (1.46–6.17)	3.37 (2.39–6.87)	0.825**

*Chi-square test.

**Kruskal Wallis test.

***Median only due to two events.

Time to recurrence = time from primary breast cancer diagnosis to recurrence diagnosis. Time to breast cancer death = time from primary breast cancer diagnosis to breast cancer-specific death.

IQR, interquartile range.

### 3.2 Recurrence and survival analyses

#### 3.2.1 Recurrence-free survival

**Figure 2:**
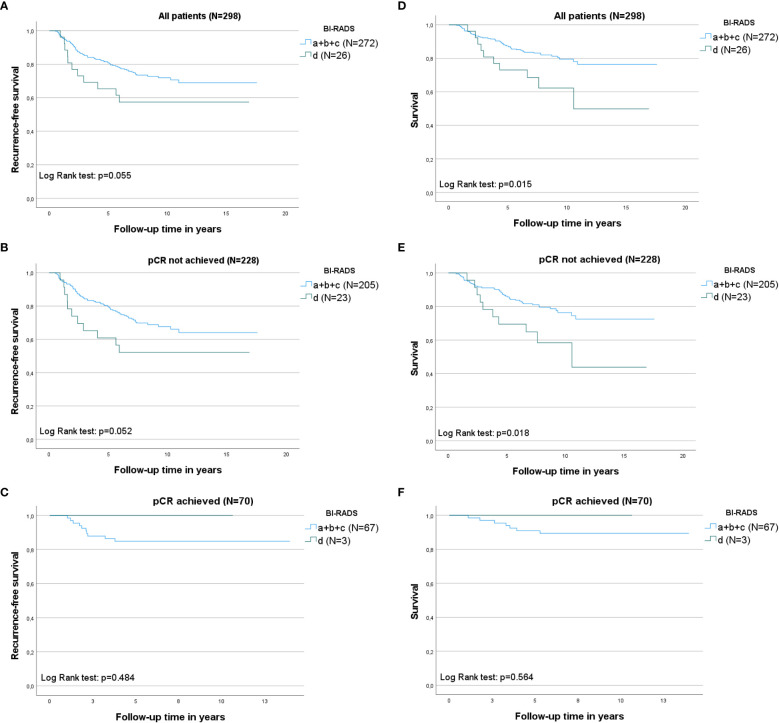
Kaplan–Meier curves illustrating recurrence-free survival and breast cancer-specific survival within the different mammographic density (MD) categories according to Breast Imaging-Reporting and Data System (BI-RADS) groups: a+b+c versus d, initially for all patients, and then split according to complete pathological response (pCR). The log-rank test was used to calculate *P* values, which can be found in each graph. N= the number of patients. Left column: Recurrence-free survival according to MD at baseline. **(A)** Recurrence-free survival in all patients; **(B)** recurrence-free survival in patients in whom pCR was not obtained; **(C)** recurrence-free survival in patients who achieved pCR. Right column: Breast cancer-specific survival (first breast cancer) according to the MD at baseline; **(D)** Breast cancer-specific survival in all patients; **(E)** breast cancer-specific survival in patients in whom pCR was not obtained; and **(F)** breast cancer-specific survival in patients who achieved pCR.

According to the KM curves (**Figure 2**, left column: A-C), patients with BI-RADS d appeared to have the shortest recurrence-free survival compared to patients with BI-RADS a/b/c (log-rank test, *P*=0.055) (please refer to section 2.3 in Methods for our interpretation of *P* values). When split per pCR, the same recurrence-free survival trends were observed in patients who did not achieve pCR (log-rank test, *P*=0.052). The findings were inconclusive for patients who achieved pCR for BI-RADS d owing to a lack of events. Patients who did not achieve pCR had shorter recurrence-free survival than those who achieved pCR.

Patients with BI-RADS d had an increased crude HR of BC recurrence vs. patients with BI-RADS a/b/c: HR 1.84 (95% CI 0.98–3.48, *P*=0.059) (**Table 3**). This association was more pronounced in the multivariate-adjusted model: HR 1.96 (95% CI 0.98–3.92, *P*=0.057). The complete multivariate model is presented in **Supplementary Material 2A**).

**Table 3:** Associations between mammographic density assessed with Breast Imaging-Reporting and Data System (BI-RADS) at diagnosis and recurrence-free survival following neoadjuvant chemotherapy.

BI-RADS	Model 1				Model 2			
	N	Events	HR (95% CI)	*P* value	N	Events	HR (95% CI)	*P* value
a+b+c	272	74	ref		254	66	ref	
d	26	11	1.84 (0.98–3.48)	0.059	25	10	1.96 (0.98–3.92)	0.057

Model 1: crude analysis.

Model 2: adjusted for age (years, continuous), ER (pos/neg), HER2 (pos/neg), axillary node status (N0/N+), tumor size at diagnosis (mm, continuous), and pCR (yes/no).

CI, confidence interval; ER, estrogen receptor; HR, hazard ratio; HER2, human epidermal growth factor receptor 2; mm, millimeter; N0, no axillary node engagement; N+, axillary node engagement; pCR, complete pathological response.

#### 3.2.2 Breast cancer-specific survival

KM curves showed that patients with BI-RADS d had the shortest BC-specific survival (**Figure 2**, right column D-F) compared to patients with BI-RADS a/b/c (log-rank test, *P*=0.015). When split by pCR, the same breast cancer-specific survival trends described above were observed in patients who did not achieve pCR (log-rank test, *P*=0.018). BC-specific survival was longer for patients in whom pCR was achieved compared to patients without pCR.

Patients with BI-RADS d had an increased crude HR of BC-specific death vs. patients with BI-RADS a/b/c: HR 2.26 (95% CI 1.15–4.44, *P*=0.019) (**Table 4**). This association was more pronounced in the multivariate-adjusted model (HR, 2.94; 95% CI 1.43–6.06, *P*=0.004). The complete multivariate model is shown in **Supplementary Material 2B**).

**Table 4:** Associations between mammographic density assessed with Breast Imaging-Reporting and Data System (BI-RADS) at diagnosis and breast cancer-specific death following neoadjuvant chemotherapy.

BI-RADS	Model 1				Model 2			
	N	Events	HR (95% CI)	*P* value	N	Events	HR (95% CI)	*P* value
a+b+c	272	52	ref		254	48	ref	
d	26	10	2.26 (1.15–4.44)	0.019	25	10	2.94 (1.43–6.06)	0.004

Model 1: crude analysis.

Model 2: adjusted for age (years, continuous), ER (pos/neg), HER2 (pos/neg), axillary node status (N0/N+), tumor size at diagnosis (mm, continuous), and pCR (yes/no).

CI, confidence interval; ER, estrogen receptor; HR, hazard ratio; HER2, human epidermal growth factor receptor 2; mm, millimeter; N0, no axillary node engagement; N+, axillary node engagement; pCR, complete pathological response.

KM curves and Cox models (BC recurrence and BC-specific survival) were also established with BI-RADS a/b as references, resulting in similar associations (**Supplementary Material 3, 4**).

## 4 Discussion

To the best of our knowledge, this is the first study to investigate whether MD could be used as a predictive marker for BC recurrence and BC-specific survival following NACT in BC patients, regardless of BC subtype. Our study indicates that patients with extremely dense breasts (BI-RADS category d) have the poorest recurrence-free survival and BC-specific survival following NACT among patients in all MD groups.

### 4.1 Comparison with studies of breast density and survival

Our results are in line with a study on NACT patients with hormone receptor+/HER2- BC by Rojas et al. (20), which found that extremely dense breasts (BI-RADS d) were associated with poorer disease-free survival (HR=1.7, *P*=0.024) compared to lower MD (BI-RADS category a-c). In the current study, all BC subtypes were included; however, because hormone receptor+/HER2- is the most common BC subtype (2), our results can essentially be compared to those of Rojas et al. (20). Meanwhile, a study by Moliere et al. (30) found no association between pretherapeutic breast density and recurrence in NACT patients, although using magnetic resonance imaging (MRI) instead of mammography as the density imaging method obstructed comparison. Concurrently, studies investigating the influence of MD on recurrence and survival in patients with BC, regardless of neoadjuvant/adjuvant treatment, are not in unison (26). Elsamany et al. (29) found that progression-free survival in patients with metastatic BC was poorer in patients with high and moderate MD [assessment according to Wolfe (39)] than in those with lower MD (HR=6.16, 95% CI 2.17–17.48, *P*=0.001). Although our results followed a similar trend to those of Elsamany et al. (29), the comparison is limited because of the different outcome measures and BC populations.

Taken together, the result presented in this paper supports previous data, that is, patients with high pre-therapeutic breast density might have worse long-term prognoses than patients with less dense breasts. However, larger studies and meta-analyses are warranted before breast density can be readily used in predictive models.

### 4.2 Rationale for choice of adjustment variables

Women who achieve pCR following NACT have improved survival, making pCR a surrogate marker for long-term survival (40) and recommended as an endpoint in clinical studies (41). The accomplishment of pCR, or lack thereof, is of great prognostic value in individual patients, especially for patients with HER2-positive tumors receiving HER2-targeted treatment (42). Thus, it is important to consider these confounders in the analysis of recurrence/survival, and histopathological tumor markers, along with tumor size and axillary nodal status, were included as adjustment variables. In this study, while tumors in high MD breasts (BI-RADS c/d) were more often ER/PR positive compared to those in low MD breasts (BI-RADS a/b), the proportion of accomplishment of pCR and HER2 status, respectively, were similar between the BI-RADS groups. Concordantly, there was no difference in HER2-targeted treatment between patients in the different BI-RADS groups. In all BI-RADS groups, when KM curves were split according to pCR, it was shown that patients who did not achieve pCR had lower recurrence-free and BC-specific survival compared to patients who achieved pCR, which is in line with other evidence suggesting that failure to achieve pCR mediates a worse prognosis (40). However, no conclusion can be drawn from patients who achieved pCR in BI-RADS category d, since no recurrences or deaths occurred in this group.

### 4.3 Mammographic density on a cellular level

Although high MD has been shown to increase the risk of BC, the exact mechanisms are not yet known (43). Breasts with high MD contain more stroma and collagen in a more linearized structure than less dense breasts (44, 45), resulting in a stiff cancer-promoting environment (43). Additionally, high density is associated with an increase in growth factors associated with fibroblasts, proteoglycans, and local production of estrogen as well as mitogens such as insulin-like growth factor I (46). Dense breast tissue and BC appear to have many similarities in the amount and type of inflammatory proteins, including Interleukin-6 and Interleukin-8, as well as increased vascular endothelial growth factor (47), possibly resulting in a pro-inflammatory microenvironment prone to carcinogenesis (48, 49).

The association between extremely dense breasts (BI-RADS category d) and recurrence/survival remained in our study despite adjusting for confounders (age at diagnosis, tumor size, pCR, axillary node status, ER status, and HER2 status), suggesting that the observed associations could be attributed to MD with a similar biological explanation as in cancer initiation in dense breasts. The observed higher recurrence and BC-specific death rates for those with tumors originating from extremely dense breasts, indicate that MD indicate that MD may have additional biological explanations compared to the aforementioned variables [e.g., large tumor size, involvement of axillary nodes, and negative hormone receptors (50)].

Our results indicate that patients with heterogeneously dense MD (BI-RADS category c) might have marginally better recurrence-free and BC-specific survival following NACT than patients with non-dense breasts (BI-RADS category a/b). This was not observed in other studies, which tended to merge breast densities into two groups. For instance, in the previously mentioned study by Elsamany et al. (29), the two groups “moderate/high MD” and “low MD” had 30 patients each. Since the inter- and intra-radiologist agreement is lowest between adjacent BI-RADS categories, highest discordant combinations between BI-RADS a and b closely followed by BI-RADS b and c (51), a separate study of BI-RADS category d may render a more selected high-MD patient group. The dichotomization of the four BI-RADS groups (category a/b vs. category c/d) in this study would likely conceal our findings of the patients with extremely high MD.

### 4.4 Future perspective

In this study, we observed that patients with extremely dense breasts (BI-RADS d) had shorter BC-specific survival. If our results were to be confirmed in larger studies, MD could be used to identify patients who might benefit from additional treatment with NACT and patients who would need intensified follow-up with the goal of possibly preventing recurrence. This could lead to more personalized treatment regimens, reduced risk of recurrence, and improved patient survival. Moreover, it may be possible to de-escalate treatment for patients with less dense breasts and to avoid unnecessary complications from overtreatment with chemotherapy. In addition to MD, as either a categorical or continuous measure, the range of imaging biomarkers associated with BC risk in mammography, breast tomosynthesis, MRI, and ultrasound would be interesting to explore in the NACT setting (52).

### 4.5 Methodological considerations

One radiologist blindly assessed the MD, and only two people reviewed the medical charts, thus minimizing the risk of information bias. The BI-RADS distribution in this study was similar to that of the Western world, where minorities of women have the most (BI-RADS d) and least dense (BI-RADS a) breasts, respectively (53), as the study population was heterogeneous due to the inclusion of both pre- and postmenopausal women and tumors of different subtypes, increasing generalizability despite this being a single-center study. Patients were identified using the Swedish Cancer Registry, to which healthcare must be reported by law, and only eight patients did not wish to participate in the study. Selection bias was considered insignificant. Through a thorough medical chart review, we were able to minimize missing values.

In the cohort, only 16 and 26 patients were found in BI-RADS groups a and d, respectively, resulting in few events in these groups. However, our results suggest strong associations despite the small sample size. We adjusted for tumor biomarkers, such as hormone receptors; however, because of the small sample size, no subgroup analyses were performed according to molecular BC subtypes. Despite these limitations, we believe these findings will be of great clinical value for further investigation.

In this observational study, pre-NACT MRI was not a clinical routine and was therefore not performed. We acknowledge the possibility of additional sites of malignancy not being identified before NACT in patients with extremely dense MD. However, in this study, >90% of patients with extremely dense MD underwent mastectomies, thus limiting this potential source of bias in our study. In this cohort, only pre-NACT MD was assessed.

Addressing longitudinal changes in MD assessed using BI-RADS, in both pre- and postmenopausal women undergoing consecutive mammographic screenings, an increase in MD augmented future BC risk, whereas a decrease in MD was associated with a lower risk (54). A potential change in MD during BC treatment (pre- and post-NACT) in relation to important clinical outcomes would be of interest to explore. In another cohort of women with BC, our group previously investigated changes in MD during NACT, concluding that most patients decreased their MD during the six cycles of NACT (24). Furthermore, in the adjuvant setting, previous studies have shown that a decrease in MD is associated with a reduced risk of contralateral BC and improved long-term survival, respectively (55, 56). In the NACT setting, changes in MD and its association with long-term survival are, to the best of our knowledge, not studied and would be an important future research goal.

## 5 Conclusion

Patients with the extremely dense breasts (BI-RADS d) were observed to have poorer recurrence-free and BC-specific survival than those with less dense breasts. This raises questions regarding individualized follow-up for women with BC with high-density breasts treated with NACT. More extensive studies are needed to confirm our findings before clinical implementation.

## Data availability statement

The datasets presented in this article are not readily available because of privacy or ethical restrictions. Requests to access the datasets should be directed to ida.skarping@med.lu.se.

## Ethics statement

The studies involving human participants were reviewed and approved by The Regional Ethics Committee of Lund, Sweden (Committee reference No.: 2014/13 and 2016/521). Written informed consent for participation was not required for this study in accordance with the national legislation and the institutional requirements.

## Author contributions

AZ collected recurrence/survival data from medical charts, performed statistical survival analyses under supervision, critically interpreted data and results, and drafted the manuscript. HS participated in data interpretation and revised the manuscript. MF participated in data interpretation and revised the manuscript. IS is the principal contributor to this study. IS collected baseline data from medical charts, performed statistical descriptive analyses, supervised the statistical survival analyses, critically interpreted the data and results, drafted the manuscript, and organized its revision. All authors contributed to the article and approved the submitted version.
