# Supplementary material for: Mammographic density in relation to breast cancer recurrence and survival in women receiving neoadjuvant chemotherapy
Source: Front Oncol. 2023 Jun 14;13:1177310. doi: 10.3389/fonc.2023.1177310 (PMC10304818; doi:10.3389/fonc.2023.1177310)
Supplement: Supplementary file 1 [file DataSheet_1.pdf]

Supplementary Material 1. Adjuvant treatment (post-surgery) according to mammographic density, assessed with Breast Imaging-Reporting and Data System (BI-RADS), at diagnosis.

		BI-RADS a	BI-RADS b	BI-RADS c	BI-RADS d	*P* value
Number of patients		16	120	140	26	
Chemotherapy	Yes	1 (6.3)	3 (2.5)	4 (2.9)	1 (3.8)	0.861*
	No	15 (93.8)	116 (96.7)	136 (97.1)	25 (96.2)	
	Missing		1 (0.8)			
HER2-targeted therapy	Yes	4 (25.0)	43 (35.8)	43 (30.7)	8 (30.8)	0.688*
	No	12 (75.0)	75 (62.5)	97 (69.3)	18 (69.2)	
	Missing		2 (1.7)			
Radiation therapy	Yes	14 (87.5)	110 (91.7)	134 (95.7)	22 (84.6)	0.157*
	No	2 (12.5)	9 (7.5)	6 (4.3)	4 (15.4)	
	Missing		1 (0.8)			
Endocrine therapy	> 5 years	3 (18.8)	38(31.7)	52 (37.1)	8 (30.8)	0.099*
	~ 5 years	1 (6.3)	13 (10.8)	22 (15.7)	5 (19.2)	
	< 5 years	2 (12.5)	22 (18.3)	17 (12.1)	8 (30.8)	
	No	10 (62.5)	46 (38.3)	49 (35.0)	5 (19.2)	
	Missing		1 (0.8)			

*Chi-square test

Abbreviation: HER2, human epidermal growth factor receptor 2

Supplementary Material 2 A). Associations between mammographic density assessed with Breast Imaging-Reporting and Data System (BI-RADS) at diagnosis and **recurrence-free survival** following neoadjuvant chemotherapy – all variables in the multivariate model (**Table 3**, model 2) – BI-RADS a+b+c *versus* d

	HR (95% CI)	*P* value
Age (years, continuous)	1.006 (0.987-1.026)	0.523
Estrogen receptor status (positive ref)	1.159 (0.688-1.953)	0.578
HER2 status (positive ref)	2.187 (1.198-3.992)	0.011
Tumor size at diagnosis (mm, continuous)	0.998 (0.991-1.005)	0.506
Axillary node status (N+ ref)	0.857 (0.512-1.433)	0.556
Complete pathological response (yes ref)	2.413 (1.105-5.273)	0.027
Mammographic density		
- BI-RADS a+b+c	ref	ref
- BI-RADS d	1.958 (0.980-3.916)	0.057

Abbreviation: HER2, human epidermal growth factor receptor 2

Supplementary Material 2 B). Associations between mammographic density assessed with Breast Imaging-Reporting and Data System (BI-RADS) at diagnosis and **breast cancer specific death** following neoadjuvant chemotherapy – all variables in the multivariate model (**Table 4**, model 2) – BI-RADS a+b+c *versus* d

	HR (95% CI)	*P* value
Age (years, continuous)	1.012 (0.991-1.034)	0.267
Estrogen receptor status (positive ref)	1.633 (0.919-2.903)	0.095
HER2 status (positive ref)	2.433 (1.197-4.948)	0.014
Tumor size at diagnosis (mm, continuous)	0.998 (0.990-1.006)	0.635
Axillary node status (N+ ref)	0.752 (0.417-1.358)	0.345
Complete pathological response (yes ref)	2.663 (1.076-6.589)	0.034
Mammographic density		
- BI-RADS a+b+c	ref	ref
- BI-RADS d	2.938 (1.425-6.058)	0.004

Abbreviation: HER2, human epidermal growth factor receptor 2

Time to breast cancer recurrence **Time to breast cancer specific death**

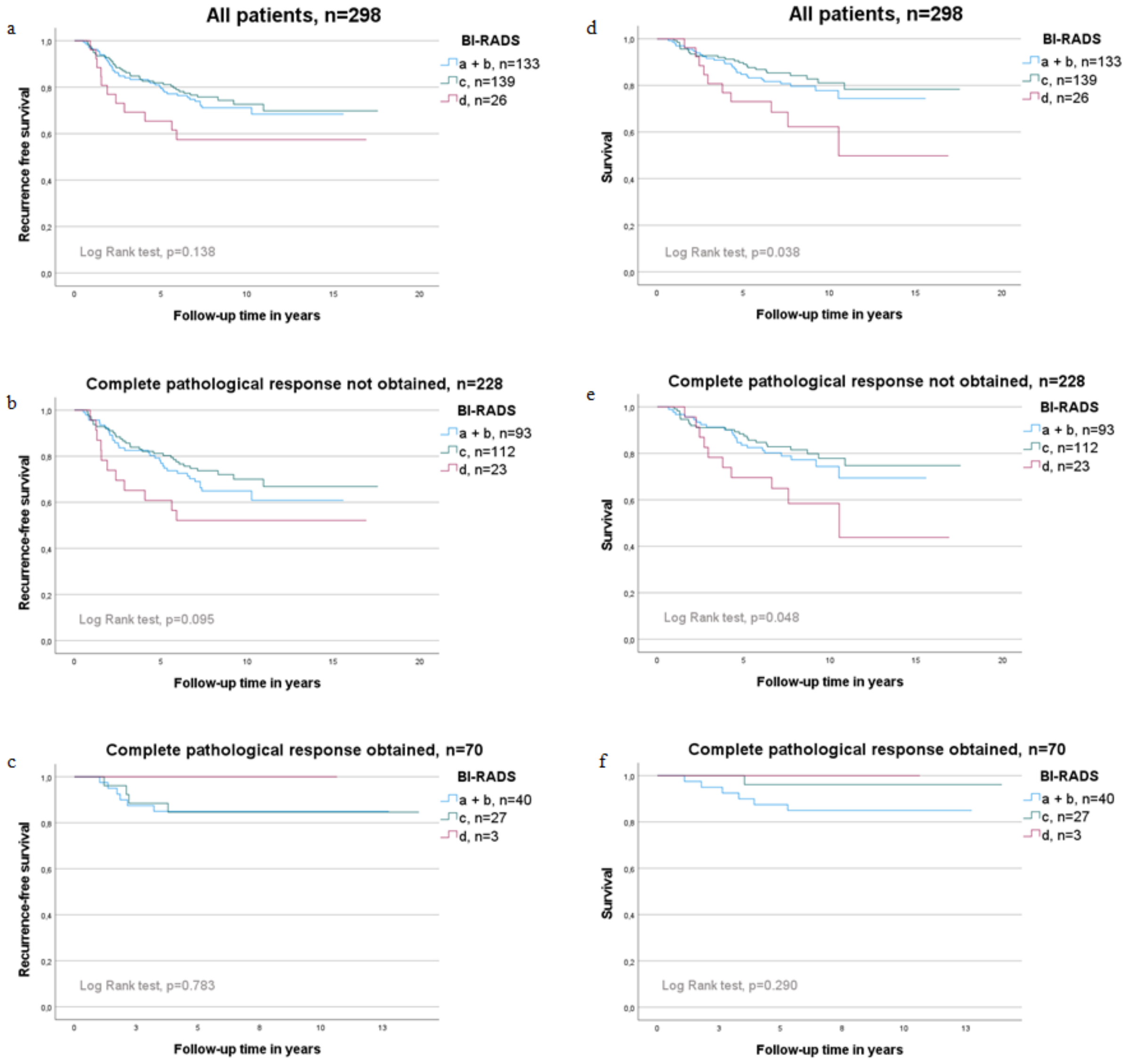

Supplementary Material 3. Kaplan–Meier curves illustrating recurrence-free survival and breast cancer-specific survival within the different mammographic density (MD) categories according to Breast Imaging-Reporting and Data System (BI-RADS) groups: a+b versus c and d, respectably. Initially for all patients, and then split according to complete pathological response (pCR). The log-rank test was used to calculate *P* values, which can be found in each graph. n = the number of patients.

Left column: Recurrence-free survival according to MD at baseline. **a)** Recurrence-free survival in all patients; **b)** recurrence-free survival in patients in whom pCR was not obtained; **c)** recurrence-free survival in patients who achieved pCR. **Right column**: Breast cancer-specific survival (first breast cancer) according to the MD at baseline; **d)** Breast cancer-specific survival in all patients; **e)** breast cancer-specific survival in patients in whom pCR was not obtained; and **f)** breast cancer-specific survival in patients who achieved pCR.

Supplementary Material 4 A). Associations between mammographic density assessed with Breast Imaging-Reporting and Data System (BI-RADS) at diagnosis and **recurrence-free survival** following neoadjuvant chemotherapy.

	Model 1				Model 2			
BI-RADS	N	Events	HR (95% CI)	*P* value	N	Events	HR (95% CI)	*P* value
a+b	133	38	ref		122	33	ref	
c	139	36	0.88 (0.56 – 1.39)	0.593	132	33	0.80 (0.48 – 1.32)	0.380
d	26	11	1.73 (0.89 – 3.39)	0.109	25	10	1.72 (0.82 – 3.64)	0.153

Model 1: Crude analysis

Model 2: adjusted for age (years, continuous), ER (pos/neg), HER2 (pos/neg), axillary node status (N0/N+), tumor size at diagnosis (mm, continuous), pCR (yes/no).

Abbreviations: CI, confidence interval; ER, estrogen receptor; HR, hazard ratio; HER2, human epidermal growth factor receptor 2; mm, millimeter; N0, no axillary node engagement; N+, axillary node engagement; pCR, complete pathological response

---

Supplementary Material 4 B). Associations between mammographic density assessed with Breast Imaging-Reporting and Data System (BI-RADS) at diagnosis and **breast cancer-specific death** following neoadjuvant chemotherapy.

	Model 1				Model 2			
BI-RADS	N	Events	HR (95% CI)	*P* value	N	Events	HR (95% CI)	*P* value
a+b	133	28	ref		122	26	ref	
c	139	24	0.79 (0.46 – 1.36)	0.391	131	22	0.68 (0.38 – 1.22)	0.195
d	26	10	2.01 (0.97 – 4.14)	0.059	25	10	2.37 (1.08 – 5.20)	0.032

Model 1: Crude analysis

Model 2: adjusted for age (years, continuous), ER (pos/neg), HER2 (pos/neg), axillary node status (N0/N+), tumor size at diagnosis (mm, continuous), pCR (yes/no)

Abbreviations: CI, confidence interval; ER, estrogen receptor; HR, hazard ratio; HER2, human epidermal growth factor receptor 2; mm, millimeter; N0, no axillary node engagement; N+, axillary node engagement; pCR, complete pathological response
